# The hidden societal cost of antibiotic resistance per antibiotic prescribed in the United States: an exploratory analysis

**DOI:** 10.1186/s12879-016-1990-4

**Published:** 2016-11-08

**Authors:** Constantinos I. Michaelidis, Michael J. Fine, Chyongchiou Jeng Lin, Jeffrey A. Linder, Mary Patricia Nowalk, Ryan K. Shields, Richard K. Zimmerman, Kenneth J. Smith

**Affiliations:** 1 Division of General Medicine and Primary Care, Brigham and Women’s Hospital, Harvard Medical School, Boston, MA USA; 2 Division of General Internal Medicine, University of Pittsburgh School of Medicine, Pittsburgh, PA USA; 3 Center for Health Equity Research and Promotion, VA Pittsburgh Healthcare System, Pittsburgh, PA USA; 4 Department of Family Medicine, University of Pittsburgh School of Medicine, Pittsburgh, PA USA; 5 PGY-3, Internal Medicine, Brigham and Women’s Hospital, 75 Frances Street, Boston, MA 02115 USA

**Keywords:** Societal costs, Antibiotic resistance, Primary care, Stewardship, Negative externality

## Abstract

**Background:**

Ambulatory antibiotic prescribing contributes to the development of antibiotic resistance and increases societal costs. Here, we estimate the hidden societal cost of antibiotic resistance per antibiotic prescribed in the United States.

**Methods:**

In an exploratory analysis, we used published data to develop point and range estimates for the hidden societal cost of antibiotic resistance (SCAR) attributable to each ambulatory antibiotic prescription in the United States. We developed four estimation methods that focused on the antibiotic-resistance attributable costs of hospitalization, second-line inpatient antibiotic use, second-line outpatient antibiotic use, and antibiotic stewardship, then summed the estimates across all methods.

**Results:**

The total SCAR attributable to each ambulatory antibiotic prescription was estimated to be $13 (range: $3–$95). The greatest contributor to the total SCAR was the cost of hospitalization ($9; 69 % of the total SCAR). The costs of second-line inpatient antibiotic use ($1; 8 % of the total SCAR), second-line outpatient antibiotic use ($2; 15 % of the total SCAR) and antibiotic stewardship ($1; 8 %). This apperars to be an error.; of the total SCAR) were modest contributors to the total SCAR. Assuming an average antibiotic cost of $20, the total SCAR attributable to each ambulatory antibiotic prescription would increase antibiotic costs by 65 % (range: 15–475 %) if incorporated into antibiotic costs paid by patients or payers.

**Conclusions:**

Each ambulatory antibiotic prescription is associated with a hidden SCAR that substantially increases the cost of an antibiotic prescription in the United States. This finding raises concerns regarding the magnitude of misalignment between individual and societal antibiotic costs.

## Background

Antibiotic resistance is a major public health problem, with more than 2,000,000 infections and 23,000 deaths caused by antibiotic-resistant organisms annually in the United States [1]. Most antibiotic use occurs in the ambulatory setting where it contributes to the development of antibiotic resistance [2, 3], which in turn increases health care costs [1, 4–7].

The relationship between ambulatory antibiotic prescribing, antibiotic resistance and increased health care costs is complex and likely influenced by heterogeneity in antibiotic use, bacterial response to antibiotic pressure, geographic distribution of resistance genes, non-human reservoirs of antibiotic resistance and impact of bacterial resistance on clinical outcomes. At a fundamental level, however, the association between ambulatory antibiotic prescribing, antibiotic resistance and increased health care costs implies a downstream societal cost of antibiotic resistance (SCAR) attributable to each ambulatory antibiotic prescription. The SCAR represents a hidden cost that, to our knowledge, has not been estimated at the prescription level and could (1) help quantify the economic burden of antibiotic resistance linked to ambulatory antibiotic prescribing, (2) inform future cost and cost-effectiveness analyses pertaining to ambulatory antibiotic prescribing, and (3) frame policy discussions regarding stewardship efforts.

Antibiotic resistance increases societal costs by increasing health care utilization, shifting antibiotic use towards more costly second-line agents, and leading to the development of antibiotic stewardship programs [1, 2]. In this exploratory analysis, we estimated the total SCAR attributable to each ambulatory antibiotic prescription in the U.S. by assuming that the total SCAR is the sum of the incremental societal costs of hospitalization, second-line inpatient antibiotic use, second-line outpatient antibiotic use, and antibiotic stewardship associated with antibiotic resistance. The goal of this exploratory analysis was to provide an initial estimate of the total annual incremental SCAR in the U.S. attributable to each ambulatory antibiotic prescription that was simple, valid and conservative, while accounting for uncertainty in sensitivity analyses. The goal of this analysis was not to quantify all potential downstream costs and benefits of ambulatory antibiotic prescribing but rather to investigate and estimate poorly understood downstream SCAR attributable to ambulatory antibiotic prescribing,

## Methods

We developed four methods to estimate the components of the SCAR attributable to each antibiotic prescription, with each method focusing on a different mechanism by which antibiotic resistance increases societal costs. These four methods estimated the incremental costs of antibiotic resistance associated with (1) hospitalization, (2) second-line inpatient antibiotic use, (3) second-line outpatient antibiotic use, and (4) antibiotic stewardship. For all methods, we estimated the total annual incremental SCAR relative to a hypothetical “no antibiotic resistance” base case scenario.

Given the complex and poorly understood relationship between ambulatory antibiotic prescribing and increased health care costs and the exploratory nature of this modeling analysis, we assumed that each human ambulatory antibiotic prescription contributed equally to the total incremental costs of antibiotic resistance associated with the four methods mentioned above and widely varied all model parameters in sensitivity analyses. If broad-spectrum antibiotics contribute more than narrow-spectrum antibiotics to the downstream social costs of ambulatory antibiotic prescriptions, this assumption would potentially over-estimate the downstream SCAR for narrow-spectrum antibiotics and under-estimate the downstream SCAR for broad-spectrum agents. To estimate this impact, we performed a secondary sensitivity analysis in which we arbitrarily assumed that broad-spectrum antibiotics were associated with double the impact of narrow-spectrum antibiotics on the total incremental SCAR attributable to each antibiotic prescription. For each method, we estimated the annual SCAR in the U.S. attributable to the specific cost mechanism of interest and then divided this estimate by the annual number of ambulatory antibiotic prescriptions in the U.S. (263 million, range: 213–263 million) [8], after adjusting for the percentage, by weight, of antibiotics prescribed to animals (80 %, range: 75–85 %) [9], the relative weighting of antibiotic use in humans versus animals on human antibiotic resistance costs (1:1, range: 1:1–3:1), and the percentage of antibiotics prescribed to humans in the inpatient setting (20 %; range: 10–30 %) [3]. We adjusted for antibiotic use in animals in light of the evidence linking such use to infections with antibiotic-resistant pathogens in humans [10]. In the absence of data, we assumed that human and animal antibiotic use had equal impact on human antibiotic resistance costs, an assumption that biases the analysis towards lower cost estimates. Within each method, we varied all parameters across wide ranges in sensitivity analyses. All costs are reported in 2013 US$ with prior years’ costs inflated using the U.S. Consumer Price Index.

We then summed the individual cost components to obtain an estimate for the total SCAR attributable to each ambulatory antibiotic prescription. This approach is conservative because it assumes that all societal costs of antibiotic resistance are captured in these four estimates.

### SCAR Component #1: Hospitalization Costs

Using a previously described approach [11], we estimated the SCAR attributable to each antibiotic prescription arising from the incremental societal costs of hospitalizations for antibiotic-resistant infections relative to antibiotic-susceptible infections (Table 1). Briefly, this approach allocates a prior estimate of the total U.S. annual incremental hospitalization cost due to antibiotic resistant infections ($41 billion, range $6–60 billion) [12–14] across the total annual number of ambulatory antibiotic prescriptions in the U.S. (263 million, range: 213–263 million) [8] after adjusting for animal and inpatient antibiotic use as described above. Although initially used to estimate the SCAR attributable to antibiotic use for acute respiratory tract infections, this approach can be generalized to all infections because it assumes equal impact of each ambulatory antibiotic prescription on societal costs of hospitalization for antibiotic-resistant infections. In the base case, we assumed that the annual incremental SCAR due to hospitalizations for antibiotic-resistant infections in the U.S. equaled $41 billion in 2013 US$ based on an estimate of annual hospitalization costs attributable to antibiotic-resistant infections relative to no infections in adults at a Chicago teaching hospital [12] extrapolated to a national level [13]. This prior study used multiple linear regression methods to estimate the incremental costs of antibiotic-resistant infections relative to no infections in a hospitalized adult cohort after controlling for age, illness severity and exposure to intensive care unit, surgery and hospital-acquired infection [12]. The study included both community and hospital-acquired antibiotic resistant infections. The range ($6–60 billion) around this point estimate represents a wide and conservative estimate based on reported result variation [12].

**Table 1:** Parameters used to estimate the hidden societal cost of antibiotic resistance (SCAR) attributable to each ambulatory antibiotic prescription in the U.S. arising from four cost components

Parameters	Base Case	Range	Source
SCAR Component #1: Hospitalization Cost			
Annual hospitalization costs due to resistant infections (US$, millions)	41,000	6,000–60,000	12–14
Percentage of annual hospitalization costs due to incremental costs			
of antibiotic-resistant versus -susceptible infections (%)	35	25–45	15,16
SCAR Component #2: Second-Line Inpatient Antibiotic Cost			
Annual antibiotic costs for resistant infections (US$, millions)			
Linezolid	743	594–892	17
Daptomycin	713	570–856	18
Carbapenems	474	379–569	19
Vancomycin	292	234–350	19
Tigecycline	148	118–178	20
Ceftaroline	88	58–118	21
Annual U.S. antibiotic costs for susceptible infections as			
a percentage of costs for resistant infections (%)	30	10–50	22
SCAR Component #3: Second-Line Outpatient Antibiotic Cost			
Antibiotic mix in the resistance scenario (%)			
Narrow-spectrum penicillins	20	16–24	23,24
First-generation cephalosporins	9	7–11	23,24
Sulfonamides	8	5–11	23,24
Tetracyclines	6	5–7	23,24
Macrolides	20	17–23	23,24
Quinolones	17	15–19	23,24
Broad-spectrum cephalosporins	9	8–10	23,24
Broad-spectrum penicillins	9	8–10	23,24
Lincomycin derivatives	2	1–3	23,24
Antibiotic mix in the no resistance scenario (%)			
Narrow-spectrum penicillins	32	30–34	23,24 Estimate
First-generation cephalosporins	14	12–16	23,24 Estimate
Sulfonamides	13	11–15	23,24 Estimate
Tetracyclines	9	7–11	23,24 Estimate
Macrolides	32	28–36	23,24 Estimate
Average cost per antibiotic class (US$)			
Narrow-spectrum penicillins (penicillin)	10	4–40	26, Estimate
First-generation cephalosporins (cephalexin)	15	4–40	26, Estimate
Sulfonamides (trimethoprim-sulfamethoxazole)	10	4–40	26, Estimate
Tetracyclines (doxycycline)	20	5–100	26, Estimate
Macrolides (azithro, clarithro and erythromycin)	25	10–200	26, Estimate
Quinolones (cipro, levo and moxifloxacin)	25	4–200	26, Estimate
Broad-spectrum cephalosporins (cefuroxime, cefdinir)	50	25–200	26, Estimate
Broad-spectrum penicillins (amoxicillin-clavulanate)	120	90–150	26, Estimate
Lincomycin derivatives (clindamycin)	50	20–80	26, Estimate
SCAR Component #4: Antibiotic Stewardship Cost			
Acute care hospital beds, U.S. (thousands)	924	Not varied	28
Staff full time equivalents, stewardship program per 200 beds			
Pharmacist	1.0	0.3–2.0	29
Physician	0.25	0.0–1.0	29
Annual salary ($US)			
Pharmacist, Infectious Diseases	120,000	100,000–140,000	30
Physician, Infectious Diseases	190,000	150,000–230,000	31
Salary multiplier for fringe benefits (%)	120	110–130	Estimate
Annual educational costs per 200 beds ($US)	15,000	10,000–20,000	Estimate
SCAR Components #1-4			
Antibiotics prescribed to humans, by weight (%)	20	15–25	9
Relative weighting of human versus animal antibiotic use	1:1	1:1–3:1	Estimate
on human antibiotic resistance costs			
Antibiotics prescribed in ambulatory setting (%)	80	70–90	3
Annual ambulatory antibiotic prescriptions (millions)	263	213–313	8

Because this prior study estimated the total incremental societal cost of hospitalization for antibiotic-resistant infections relative to no infections rather than relative to antibiotic-susceptible infections, it potentially overestimates the incremental costs of antibiotic-resistant infections relative to antibiotic-susceptible infections. To correct for this potential overestimate, we assumed that only 35 % (range: 25–45 %) of the total incremental societal costs of hospitalization for antibiotic-resistant infections could be attributable to antibiotic resistance based on estimates of the incremental costs of antibiotic-resistant versus antibiotic-susceptible Staphylococcal and Enterococcal hospital infections [15, 16].

### SCAR Component #2: Second-Line Inpatient Antibiotic Costs

To estimate the SCAR attributable to each antibiotic prescription arising from the incremental cost of second-line inpatient antibiotic use, we assumed that this cost equaled the total annual incremental U.S. cost of antibiotics used, in the inpatient setting, primarily for the treatment of antibiotic-resistant infections (carbapenems, ceftaroline, daptomycin, linezolid, tigecycline and vancomycin) relative to antibiotics used primarily for the treatment of antibiotic-susceptible infections (Table 1) [17–21]. The above antibiotics were selected by authors in an effort to select those agents most likely to be used in the setting of concern for antibiotic resistance and informed by a discussion of recent antibiotic development [1]. We conservatively did not include the aminoglycosides given some concern that utilization may not predominantly reflect concern for resistance. Given that inpatient aminoglycoside use accounts for only 1.8 % of total inpatient antibiotic use and $71 million in annual costs, this modeling choice would be unlikely to materially impact results [17]. Annual U.S. antibiotic cost data were drawn from the IMS Health National Sales Perspective database [17, 19] and manufacturer quarterly financial reports [18, 20, 21]. We assumed that 100 % of the annual cost of each of the aforementioned antibiotics and 0 % of the annual cost of all other antibiotics were associated with the treatment of antibiotic-resistant infections in the inpatient setting. We assumed that antibiotic-susceptible infections would have occurred in the absence of antibiotic resistant-infections and that antibiotic costs for treatment of antibiotic-susceptible infections were 30 % (range: 10–50 %) of antibiotic costs for treatment of resistant infections based on decreased overall antibiotic costs for patients hospitalized with methicillin-susceptible versus methicillin-resistant Staphylococcus aureus infections [22]. We did not consider antibiotic development costs in our estimates due to concerns that doing so could result in double-counting of societal costs.

### SCAR Component #3: Second-Line Outpatient Antibiotic Costs

To estimate the SCAR attributable to each antibiotic prescription arising from the incremental societal costs of second-line outpatient antibiotic use, we assumed this cost was a function of incremental antibiotic costs in the presence versus absence of antibiotic resistance (Table 1). In both the “resistance” and “no resistance” scenarios, we assumed 263 million (range: 213–313 million) ambulatory antibiotic prescriptions annually in the U.S. based on IMS Health pharmacy-level data for dispensed antibiotic prescriptions [8]. To define the antibiotic mix in the “resistance” scenario, we assumed that the current mix of narrow- and broad-spectrum antibiotics reported in the NAMCS/NHAMCS data for pediatric [23] and adult [24] populations reflects physician prescribing behavior in the setting of concern for antibiotic resistance. To define the antibiotic mix in the “no resistance” scenario, we assumed that use of broad-spectrum antibiotics (broad-spectrum penicillins and cephalosporins, quinolones and lincomycin derivatives) reflects concern for antibiotic-resistant pathogens and would not have occurred in the absence of antibiotic resistance. In this scenario, we assumed that 100 % of broad-spectrum antibiotics would be replaced by narrow-spectrum antibiotics (narrow-spectrum penicillins, first-generation cephalosporins, macrolides, sulfonamides, tetracyclines) in the same volume-weighted ratios reported in the NAMCS/NHAMCS data [23, 24]. Using a previously described approach [25], we estimated antibiotic costs using prices from an online pharmacy database [26] and not average wholesale prices (AWPs) due to concerns that AWPs do not accurately reflect acquisition costs [27]. Because this online pharmacy database only includes costs for antibiotics commonly prescribed in the ambulatory setting, we were unable to use this database for the second-line inpatient antibiotic cost estimation method described above. For each antibiotic class, we assumed that the class included only commonly used agents within that class (Table 1).

We assumed that antibiotic resistance caused only an increase in cost per ambulatory antibiotic prescription and not an increase in the number of ambulatory antibiotic prescriptions per patient for a given infection or total duration of antibiotic therapy. This assumption is likely conservative given that ambulatory patients with antibiotic-resistant pathogens may return to clinic more frequently than those with antibiotic-susceptible pathogens and require multiple antibiotic prescriptions [7].

### SCAR Component #4: Antibiotic Stewardship Costs

To estimate the SCAR attributable to each antibiotic prescription arising from the incremental societal costs of antibiotic stewardship, we assumed that such costs would not have been incurred in the absence of concern for the potential development of antibiotic resistance and equaled the annual costs of antibiotic stewardship programs incurred by U.S. hospitals (Table 1). Given an absence of published literature providing detailed cost data for such programs, we extrapolated the annual costs of antibiotic stewardship programs from the annual costs of antibiotic stewardship programs per acute care bed [28, 29]. Based on guidelines from the Infectious Disease Society of America, we assumed that the salary costs for infectious disease trained physicians (0.25 full time equivalent per 200 beds, range: 0.0–1.0) and pharmacists (1.0 full time equivalent per 200 beds, range: 0.3–2.0) represented the primary costs of such programs [29–32]. In addition, because educational activities are a core element of antibiotic stewardship programs, we estimated annual costs of educational activities of $15,000 (range: $10,000–20,000) per 200 beds, assuming that 30 physicians would attend 24-h educational activities annually at an average salary cost of $120 per hour [31]. This estimate is conservative in that it does not include costs of collaboration with clinical microbiologists, infection control officers, hospital epidemiologists, or information technology specialists [32]. Furthermore, this estimate does not include physician time costs incurred when interfacing with antibiotic stewardship programs that include formulary restriction and pre-authorization as core program components [32].

## Results

When we summed the costs of all four components, the SCAR attributable to each ambulatory antibiotic prescription was $13 in the base case and ranged from $3–$95 in sensitivity analyses (Table 2). The greatest contributor to the base case total SCAR estimate was the cost of hospitalization (69 %; base case: $9, range: $1–$39). The costs of second-line inpatient antibiotic use (8 %; base case: $1, range: $0–$4), second-line outpatient antibiotic use (15 %; base case: $2, range: $2–$47) and antibiotic stewardship (8 %; base case: $1, range: $0–$5) were modest contributors to the total SCAR attributable to each antibiotic prescription. Assuming an average antibiotic cost of $20, the total SCAR attributable to each ambulatory antibiotic prescription would increase antibiotic costs by 65 % (range: 15–475 %) if incorporated into antibiotic costs experienced by patients or payers.

**Table 2:** Estimate of the hidden societal cost of antibiotic resistance (SCAR) attributable to each ambulatory antibiotic prescription in the U.S. arising from four cost components

SCAR Component	Cost Estimates	
	Base Case	Range
#1: Hospitalization Cost	$9	$1–$39
#2: Second-line Inpatient Antibiotic Cost	$1	$0–$4
#3: Second-line Outpatient Antibiotic Cost	$2	$2–$47
#4: Antibiotic Stewardship Cost	$1	$0–$5
Total SCAR Attributable to Each Antibiotic Prescription^a^	$13	$3–$95

^a^Sum of the four cost components above, rounded to nearest $US

In the secondary sensitivity analysis in which we assumed that a broad-spectrum antibiotic prescription was associated with double the impact of narrow-spectrum antibiotic prescription on the total incremental SCAR attributable to each ambulatory antibiotic prescription, we estimated that the total downstream SCAR attributable to each ambulatory antibiotic prescription was $9 for a narrow-spectrum antibiotic prescription and $17 for a broad-spectrum antibiotic prescription.

Assuming that there are 263 million ambulatory antibiotic prescriptions annually in the United States [8] and that 30 % of all ambulatory antibiotic prescribing is inappropriate [33], our base case estimate for the total SCAR attributable to each ambulatory antibiotic prescription ($13) suggests that total and inappropriate ambulatory antibiotic prescribing account for $3.4 billion and $1.0 billion, respectively, in SCAR annually in the United States.

## Discussion

This exploratory analysis quantifies the hidden, but considerable, societal cost of ambulatory antibiotic prescribing. We conservatively estimated that the hidden SCAR attributable to each ambulatory antibiotic prescription is $13 in the base case and substantially increases the societal cost of an antibiotic prescription if incorporated into antibiotic costs paid by patients or payers.

This estimate has important implications for patients, clinicians, payers and policy makers. For patients and clinicians, this study further raises the costs of inappropriate antibiotic prescribing for diagnoses such as the common cold, upper respiratory tract infections, and acute bronchitis, for which antibiotics are commonly prescribed [34, 35] despite evidence that the patient benefit is virtually zero [36, 37] and of harm is moderate [38]. For conditions such as community-acquired pneumonia, skin and soft-tissue infections and urinary tract infections where antibiotics more clearly provide benefit, this study has more limited implications. For policymakers, this study raises serious concerns regarding the risks of not considering the hidden SCAR attributable to ambulatory antibiotic prescribing and the optimal mechanism to appropriately allocate this economic burden. The major risk of ignoring the SCAR attributable to each ambulatory prescription is to perpetuate a misalignment because individual and societal costs that encourages excess antibiotic use to that which would be expected if societal costs were considered in a perfect market [39, 40].

Classically, economists have proposed several solutions to address such a ‘negative externality’—regulation, taxation or permits, all of which have been considered as solutions to ease the economic burden of antibiotic resistance attributable to antibiotic use [40]. These cost allocation mechanisms, however, assume that there is a single antibiotic consumer in the U.S. healthcare system who *should* bear this economic burden. Future discussion of the appropriate bearer of this economic burden and the optimal cost allocation mechanism must be informed by an understanding of the magnitude of the economic burden and the likely impact of any cost allocation mechanism on health outcomes. This study did not attempt to quantify all potential downstream costs and benefits of ambulatory antibiotic prescribing but rather one poorly understood downstream cost of ambulatory antibiotic prescribing, namely the costs of antibiotic resistance. In estimating the economic burden of antibiotic resistance associated with ambulatory antibiotic prescribing, this analysis provides one element necessary for an important discussion regarding net costs and benefits of ambulatory antibiotic prescribing.

This study has several strengths. First, this study employed a novel approach to estimate the hidden SCAR attributable to each ambulatory antibiotic prescription. Second, this study incorporated conservative assumptions that biased the analysis in favor of lower cost estimates. Within the four estimation methods, we focused only on major cost drivers. Further, when summing across the four estimation methods, we assumed that they represented all of the mechanisms by which antibiotic resistance increases societal costs. If there are other material mechanisms by which antibiotic use leads to SCAR, i.e. by leading to increased utilization of outpatient services, then this analysis would result in a conservative estimate of the total SCAR attributable to each ambulatory antibiotic prescription.

Our analysis also has limitations. First, in the absence of published data describing the relative impact of human and agricultural antibiotic use on antibiotic resistance in humans, all four estimation methods relied on our estimate of the relative impact of human versus animal antibiotic use on antibiotic resistance costs in humans. Our assumption that each unit weight of antibiotic use in humans and animals equally impacts antibiotic-resistance costs in humans, however, is likely conservative. Second, in the absence of available data allowing us to make reasonable estimates regarding the relative contribution of different antibiotics (i.e. amoxicillin relative to amoxicillin-clavulanate or first-generation cephalosporins relative to quinolones), we assumed that each human ambulatory antibiotic prescription contributed equally to the total incremental SCAR, which may potentially over-estimate the downstream SCAR for narrow-spectrum antibiotics and under-estimate the downstream SCAR for broad-spectrum antibiotics are greater contributors to the total incremental SCAR than narrow-spectrum antibiotics. Third, in the absence of published data providing further guidance, our four estimation methods did not account for antimicrobial prescribing in long-term inpatient care facilities, potential contribution of inappropriate antibiotic dosing and duration and antibiotic resistance introduced from travel exposures outside of the United States. Fourth, each method had unique limitations. In the second-line inpatient antibiotic cost method, we estimated the SCAR attributable to ambulatory antibiotic prescribing based in part on antibiotics that are predominantly used for treatment of antibiotic resistant infections in the hospital setting (i.e. carbapenems, daptomycin, etc.). Further, some proportion of second-line antibiotic use may be inappropriate and not reflective of concern for antibiotic resistant infections, which could lead to an overestimate of the SCAR arising from this source. In the second-line outpatient antibiotic cost method, we assumed that ambulatory use of broad-spectrum antibiotics predominantly reflects physician concern for antibiotic resistance when it may also reflect other attitudes towards the value of specific antibiotics. In the antibiotic stewardship cost method, we estimated total U.S. costs of antibiotic stewardship by extrapolating costs of stewardship from a 200-bed community hospital to a national level based on bed size, assuming a linear relationship between bed-size and stewardship costs across all hospitals [28, 29]. Finally, these estimates are unlikely to be generalizable outside of the U.S. given different antibiotic utilization, resistance and costs.

## Conclusions

In summary, we found that there is a considerable hidden SCAR attributable to each ambulatory antibiotic prescription that would substantially increase the total societal costs of ambulatory antibiotic prescribing if incorporated into costs experienced by payers or patients. The magnitude of the misalignment between antibiotic costs experienced on individual and societal levels reinforces the need to develop and validate innovative strategies to appropriately align individual and societal costs of antibiotic use in an effort to safely reduce total societal costs of antibiotic use.

## Abbreviation

SCAR: Societal cost of antibiotic resistance
